# Robust topological temperature localization in thermal rock–paper–scissors chain

**DOI:** 10.1093/nsr/nwag169

**Published:** 2026-03-20

**Authors:** Zhaochen Wang, Quan Liu, Xin Qian, Xiaobing Luo, Run Hu

**Affiliations:** School of Energy and Power Engineering, Huazhong University of Science and Technology, Wuhan 430074, China; School of Energy and Power Engineering, Huazhong University of Science and Technology, Wuhan 430074, China; School of Energy and Power Engineering, Huazhong University of Science and Technology, Wuhan 430074, China; School of Energy and Power Engineering, Huazhong University of Science and Technology, Wuhan 430074, China; School of Energy and Power Engineering, Huazhong University of Science and Technology, Wuhan 430074, China; State Key Laboratory of High-Density Electrical Energy Conversion, Huazhong University of Science and Technology, Wuhan 430074, China; Department of Applied Physics, Kyung Hee University, Yongin-Si 17104, Republic of Korea; Shenzhen Institute of Huazhong University of Science and Technology, Shenzhen 518052, China

**Keywords:** temperature localization, topological phase transition, topological heat transfer, rock-paper-scissors

## Abstract

Emerging topological thermal physics has revolutionized thermal management with topological thermal metamaterials, but most of them only consider passive/static thermal diffusion. Inspired by ecological dynamics, we investigate the topological thermal physics in an active/dynamic three-body heat-transfer system connected with Peltier modules, in which the cyclic interaction is exemplified by the rock–paper–scissors (RPS) chain. Numerical simulations demonstrate a robust temperature localization phenomenon against parametric disturbance and structural perturbations, and topological phase transition in a thermal RPS chain is discussed via a topological band-theory analysis of the corresponding Hamiltonian. Our findings establish a framework for exploring dynamic topological phenomena in non-equilibrium thermal transport, offering new pathways for active thermal management.

## Teaser

Inspired by rock-paper-scissors (RPS) cycles in ecosystem, a thermal RPS model is established to reveal the robust topological temperature localization phenomenon and topological phase transition in active thermal transport system.

## INTRODUCTION

Inspired by topological photonics and topological acoustics [1–7], the counterpart topological thermal transport, enabled by thermal metamaterials [8–14], has emerged as a groundbreaking research paradigm beyond traditional heat transfer that significantly reshapes the way we think about and deal with heat, and advances thermal management technologies such as thermal shielding/cloaking and harvesting/concentrating devices [15–25]. Under such a physical framework, a lot of topological thermal physics has been reported, such as nonreciprocal/asymmetric heat transfer [26–28], skin effect [29,30], geometrical phase [31–33], protected edge state [34,35], bulk–boundary correspondence [36–38] and so on. However, most of them only consider passive/static thermal diffusion, but can we observe more thermal physics in active/dynamic systems?

**Figure 1.**
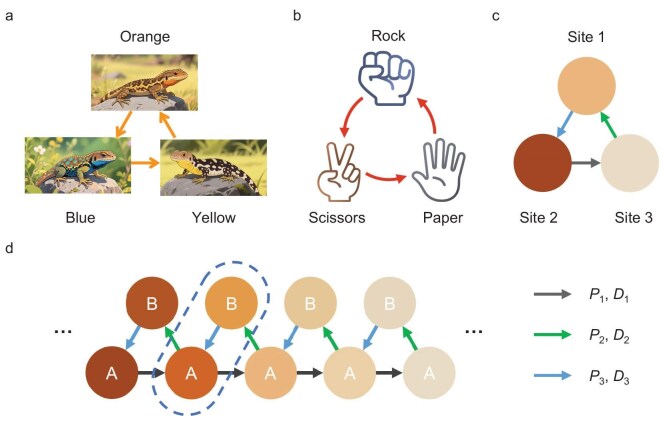
Schematic of thermal RPS chain. (a) Mutually restrictive relationships of three typical species of side-blotched lizards with orange-, blue- and yellow-colored stripes. (b) RPS game. (c) Thermal RPS cycle with different active transport coefficient *P_j_* and thermal diffusion coefficient *D_j_* values. (d) Structure of thermal RPS chain connected by a series of RPS cycles. Thermal sites A and B can be regarded as one unit cell.

Recently, active topological phase-transition systems in active matter [39–41], fluid dynamics [42,43] and ecological systems [44–47] have started to be developed, but the counterpart thermal physics in such systems remains underexplored. A typical ecological system has population dynamics in which the number of individuals in a population changes over time, affected by factors such as births, deaths, predation and competition. Real ecological systems are extremely complex, with numerous species and intricate interactions. Under specific situations, population dynamics can be simplified to only consider the interactions among three species, such as communities containing toxin-producing *Escherichia coli* [48,49], competition among cryptic coral reefs [50] and the reproductive strategies of side-blotched lizards [51]. Due to predation and competition, the population of three such species will be dynamically balanced and can be modeled by using the rock–paper–scissors (RPS) cycle. Take side-blotched lizards with differently colored stripes as an example. Orange males (analogous to ‘rock’), characterized by aggression, overpower blue males through physical superiority and expansive harems. Blue males (analogous to ‘scissors’), with smaller territories and heightened vigilance, effectively counter yellow males that mimic females and steal mating opportunities. Yellow males (analogous to ‘paper’) counter orange males by stealthily infiltrating their territories and stealing mating opportunities (Fig. 1a and b). Such an RPS model describes the cyclic competition in a lot of systems ranging from biodiversity in ecosystems [48–51] to phase transitions in non-equilibrium quantum systems [44–46]. Recent advances in topological physics have revealed that such RPS cyclic interactions can encode geometric phases and protected edge modes in synthetic lattices [44,45]. Can we extend such an RPS model to describe active/dynamic thermal physics beyond passive/static thermal diffusion?

In this letter, inspired by the RPS cycle in ecological systems, we establish an actively regulated three-body heat-transfer system connected with Peltier modules to mimic RPS cyclic interactions (Fig. 1c) and extend the RPS cycles into a RPS chain to explore the topological physics of thermal transport. Robust temperature localization due to heat accumulation is found in the RPS chain and the topological phase transitions are analysed through energy-band theory.

## RESULTS

### Generation of thermal RPS chain

To implement the RPS model in thermal diffusion systems, we connect three bodies via Peltier modules and the whole system is assumed to have the same material and structure (Fig. 1d). Heat transfer between every two bodies consists of intrinsic thermal diffusion (proportional to the temperature gradient) and Peltier heating/cooling power (proportional to the average temperature). We then connect a series of RPS cycles into an RPS chain and each unit cell consists of two thermal sites (A and B). The heat flow through the Peltier module occurs at a rate of *Q* = *SIT*_avg_, where *S, I* and *T*_avg_ are the Seebeck coefficient, the electric current and the average temperature of the channel, respectively. The heat-flow direction depends on the direction of the current. In each RPS cycle, as shown in Fig. 1b, the active transport coefficients of the three channels are *P*_1_, *P*_2_ and *P*_3_, respectively, and the corresponding intrinsic diffusion coefficients are *D*_1_, *D*_2_ and *D*_3_, respectively (detailed in Note SI). By discretizing the heat-transfer equations in the RPS chain, we can obtain the governing equations of the temperature field as:


(1) \begin{eqnarray*}
\left\{ {\begin{array}{@{}*{1}{c}@{}} \begin{array}{@{}c@{}} \frac{{\partial {T}_{n,A}}}{{\partial t}} = {D}_1\left( {{T}_{n + 1,A} - {T}_{n,A}} \right) + {D}_1\left( {{T}_{n - 1,A} - {T}_{n,A}} \right)\\ \quad+ {D}_2\left( {{T}_{n - 1,B} - {T}_{n,A}} \right) + {D}_3\left( {{T}_{n,B} - {T}_{n,A}} \right)\\ \quad+ {P}_3\left( {{T}_{n,B} + {T}_{n,A}} \right) + {P}_1\left( {{T}_{n - 1,A} + {T}_{n,A}} \right)\\ \quad- {P}_2\left( {{T}_{n - 1,B}{\mathrm{ + }}{T}_{n,A}} \right) - {P}_1\left( {{T}_{n + 1,A} + {T}_{n,A}} \right) \end{array}\\ \begin{array}{@{}c@{}} \frac{{\partial {T}_{n,B}}}{{\partial t}} = {D}_2\left( {{T}_{n + 1,A} - {T}_{n,B}} \right) + {D}_3\left( {{T}_{n,A} - {T}_{n,B}} \right)\\ \quad + {P}_2\left( {{T}_{n + 1,A} + {T}_{n,B}} \right) - {P}_3\left( {{T}_{n,A} + {T}_{n,B}} \right) \end{array} \end{array}} \right.,\!\!
\end{eqnarray*}

where *T_n,A_* and *T_n,B_* denote the temperature of site A and B in unit *n*. The discretized RPS chain system yields an effective Hamiltonian for the site-channel model, establishing a thermal analog (antisymmetric Hamiltonian) for the RPS mass-conserving dynamical system (detailed derivation in Note SI). Equation (1) can be articulated as ${\partial }_t{{\bf \hat{T}}} = - i H{{\bf\hat{T}}}$, where $${{\bf \hat{T}}} = {[ {\begin{array}{*{20}{c}} {{T}_{1,A}}&{{T}_{1,B}}& \cdots &{{T}_{n,B}} \end{array}} ]}^T$$ represents the temperature field of the whole RPS chain, *H* corresponds to the Hamiltonian operator and *i* is the imaginary unit. For theoretical analysis with periodic boundary conditions (PBCs), we close the chain by coupling the last B site back to the first A site, forming a ring with an even total number of sites *s* = 2*N*, where *N* is the total number of units. For the open boundary conditions (OBC) used in most situations, the chain is terminated, resulting in an odd total number of sites *s* = 2*N* + 1 (see detailed discussion with boundary conditions in Note SIV). In an OBC thermal RPS chain, the corresponding Hamiltonian can be written as:


\begin{eqnarray*}
{{H}} = \left( { - i} \right) \cdot \left[
{\begin{array}{@{}c@c@c@c@c@c@c@{}} {{D}_1 + {D}_3 + {P}_1 - {P}_3}&{ - {D}_3 - {P}_3}&{ - {D}_1 + {P}_1}& \cdots &{}&{}&{}\\ { - {D}_3 + {P}_3}&{{D}_2 + {D}_3 + {P}_3 - {P}_2}&{ - {D}_2 - {P}_2}& \cdots &{}&{}&{}\\ { - {D}_1 - {P}_1}&{ - {D}_2 + {P}_2}&{2{D}_1 + {D}_2 + {D}_3 + {P}_2 - {P}_3}&{ - {D}_3 - {P}_3}&{ - {D}_1 + {P}_1}& \cdots &{}\\ \vdots & \vdots &{ - {D}_3 + {P}_3}&{{D}_2 + {D}_3 + {P}_3 - {P}_2}&{ - {D}_2 - {P}_2}& \cdots &{}\\ {}&{}& \vdots & \vdots & \ddots & \vdots & \vdots \\ {}&{}&{}& \cdots &{ - {D}_3 + {P}_3}&{{D}_2 + {D}_3 + {P}_3 - {P}_{_2}}&{ - {D}_2 - {P}_2}\\ {}&{}&{}& \cdots &{ - {D}_1 - {P}_1}&{ - {D}_2 + {P}_2}&{{D}_1 + {D}_2 + {P}_2 - {P}_1} \end{array}} \right].\\
\end{eqnarray*}

Without loss of generality, we assume that *P*_1_ = *P*_3_ and define *R* = *P*_2_/*P*_3_ as the skewness parameter for exploring the thermal energy dynamics. The RPS chain can be thought of as a 1D chain of nonlinear oscillators because each isolated RPS cycle represents a local oscillator in which heat oscillates between the different sites. When *P_j_* >> *D_j_* (*j* = 1,2,3), *D_j_* in the Hamiltonian can be neglected and the Hamiltonian H becomes antisymmetric (see Note SI for details). When *D_j_* and *P_j_* are at the same order of magnitude, both will influence the topological properties significantly. In the following, intricate temperature distribution patterns depending on the skewness parameter *R* are revealed.

### Temperature localization and robustness in thermal RPS chain

**Figure 2.**
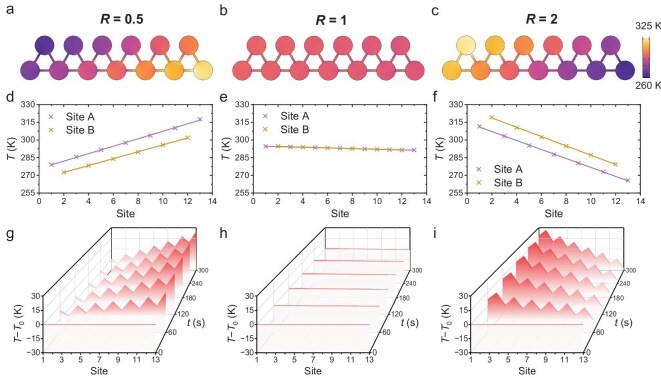
Temperature localization in thermal RPS chain (*s* = 13). (a–c) Temperature distribution of thermal RPS chain with skewness *R* = 0.5, 1 and 2. (d–f) Steady-state temperature of sites A and B in thermal RPS chain with skewness *R* = 0.5, 1 and 2, respectively. (g–i) Transient temperature change of each site in thermal RPS chain with skewness *R* = 0.5, 1 and 2, respectively.

For skewness *R* < 1, more thermal energy is moved to the right due to the smaller *P*_2_ in the RPS cycle, resulting in the maximum temperature in the right boundary of the RPS chain, which corresponds to the progressive polarization toward the right boundary over time, regardless of the initial conditions (Fig. 2a). Conversely, for *R* > 1, more thermal energy is moved to the left and a left-boundary polarization emerges (Fig. 2c). For *R* = 1, there is no-biased net thermal energy and the temperature field preserves homogeneity across the whole chain (Fig. 2b). This temperature localization manifests as a decay in the average temperature per site along the chain direction. We quantify this polarization by evaluating the average temperature of each site. The temperature profile exhibits a jagged pattern, alternating between the A and B sites. When examined separately, the temperatures of the A sites across different units show a consistent gradient, as do those of the B sites (Fig. 2d–f). Such localization arises for any initial temperature distribution and is intuitively demonstrated in a finite system (*s* = 13). Remarkably, the skewness *R* exclusively determines whether the overall temperature field polarizes to the left or the right boundaries of the chain, while the values of the other parameters only influence the temperature values and not the direction of localization.

Although the governing Hamiltonian *H* is derived from the transient Equation (1), the temperature distribution of the thermal RPS model eventually evolves toward a steady state after a certain period of time. To further investigate the transient temperature characteristic of the thermal RPS chain, the initial temperature of each site is set to the reference temperature (*T*_0_ = 293.15 K). Figure 2g–i illustrate the transient temperature distribution with skewness *R* = 0.5, 1 and 2. When *R* ≠ 1, a temperature localization effect emerges and strengthens over time. The direction of this localization remains consistent with the steady-state profile, while its magnitude increases progressively, starting from an initially uniform distribution and eventually converging to the final steady state. When *R* = 1, the heat transfer between each site forms a dynamic equilibrium. Therefore, the temperature remains constant at the initial value.

We quantify the temperature localization by calculating the temperature-weighted geometric center ${\bar{\,\,x}}_T = {{\int_{0}^{L}{{xT( x )}}dx} / {\int_{0}^{L}{{T( x )}}dx}}$, where *L* is the total length of the thermal RPS chain (see Note SII for details). When *R* = 1, the temperature is uniform and thus ${\bar{\,\,x}}_T$ coincides with the real geometric center ${\bar{\,\,x}}_g = {L / 2}$ of the RPS chain. In contrast, ${\bar{\,\,x}}_T$ shifts from ${\bar{\,\,x}}_g$ when *R* varies, which could be used for characterizing the temperature localization. When *R* < 1, ${\bar{\,\,x}}_T$ > ${\bar{\,\,x}}_g$, indicating that the overall temperature undergoes polarization to the right. When *R* > 1, ${\bar{\,\,x}}_T$ < ${\bar{\,\,x}}_g$, indicating that the overall temperature undergoes polarization to the left. The further that ${\bar{\,\,x}}_T$ deviates from ${\bar{\,\,x}}_g$, the greater the polarization strength will be.

**Figure 3.**
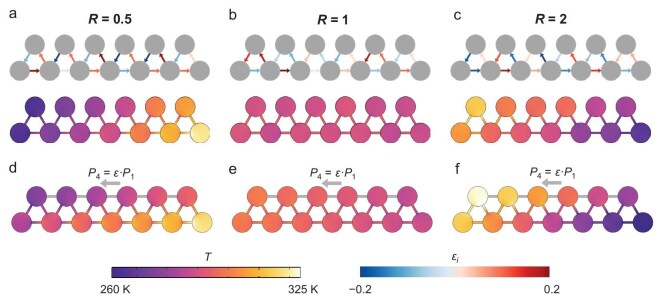
Robustness of temperature localization in thermal RPS chain. (a–c) Applied disturbance on active transport coefficient ${P}_n = {P}_n( {1 + {\varepsilon }_n} )$ and the corresponding temperature distribution. The perturbation coefficient *ε_n_* is denoted by the color of the channel. (d–f) Structural perturbations and extra couplings with active transport coefficient ${P}_4 = \varepsilon \cdot {P}_1$ and the corresponding temperature distribution.

The above temperature localization demonstrates exceptional robustness against both parametric disturbance and structural perturbations. Figure 3a–c illustrate the polarization of temperature to the boundary when the active transport coefficients are perturbed as ${P}_i = {P}_i( {1 + {\varepsilon }_i} )$, where *P_i_* is the active transport coefficient of the *i*-th channel and the perturbation *ε_i_* values are randomly chosen within the set of {*ε_i_*|−0.2 < *ε_i_* < 0.2}. It can be seen that the states of temperature localization are not affected by the perturbation *ε_i_* with different *R* values, indicating great robustness against the disturbance. On the other hand, the temperature localization also remains robust when extra coupling between each site B is introduced, just as the predator–herbivore relationships in nature are affected by external influences. Specifically, extra channels between each site B are added to the RPS chain with an active transport coefficient ${P}_4 = \varepsilon \cdot {P}_1$ (Fig. 3d–f). These additionally incorporated couplings (*ε* = 0.3) fail to disrupt the fundamental qualitative temperature localization direction.

### Topological phase transition and topological band theory

For *R* = 1, thermal energy is uniformly distributed along the chain and the temperature is homogeneous. Otherwise, any change in *R* will cause the temperature of the entire system to start polarization and eventually lead to a new state of localization. More generally, from our following calculations, it turns out that it is not possible to tune any set of active transport coefficients on the RPS chain such that one passes from localization at one boundary to the other boundary without crossing a transition point at *R* = 1. Further research on topological band theory will also support this view, as follows.

To further characterize the topological properties of the RPS chain, we apply PBC to calculate the Bloch Hamiltonian as:


\begin{eqnarray*}
{{H}}\left( k \right) = \left( { - i} \right)\left[
{\begin{array}{@{}c@{\quad}c@{}} {\left( {2{D}_1 + {D}_2 + {D}_3 - {P}_3 + {P}_2} \right) - \left( {{D}_1 - {P}_1} \right){e}^{ik} - \left( {{D}_1 + {P}_1} \right){e}^{ - ik}}&{ - \left( {{D}_3 - {P}_3} \right) - \left( {{D}_2 + {P}_2} \right){e}^{ik}}\\ { - \left( {{D}_3 + {P}_3} \right) - \left( {{D}_2 - {P}_2} \right){e}^{ - ik}}&{{D}_2 + {D}_3 - {P}_2 + {P}_3} \end{array}} \right],
\end{eqnarray*}

where *k* is the wave number in the Brillouin zone (BZ), defined as *β* = *e^ik^, k*∈(−π, π). When *P_j_* >> *D_j_*, we ignore the diffusion coefficient *D_j_* and the Hamiltonian is simplified as:


(4) \begin{eqnarray*}
{{H}}\left( k \right) = \left( { - i} \right)\left[ {\begin{array}{@{}*{2}{c}@{}} {{P}_2 - {P}_3 + {P}_1{e}^{ik} - {P}_1{e}^{ - ik}}&{{P}_3 - {P}_2{e}^{ik}}\\ { - {P}_3 + {P}_2{e}^{ - ik}}&{{P}_3 - {P}_2} \end{array}} \right].
\end{eqnarray*}

In non-Hermitian systems, a conventional BZ fails to correctly predict the spectrum and topological characteristics under OBC [5,52]. In order to analyse the topological localization phenomena, the non-Hermitian Bloch vector should be extended to the complex domain to recover the breakdown of the bulk–boundary correspondence. This implies that the usual Bloch phase factor *e^ik^* is generalized to *β* = *e^ik^^’^* = *re^ik^* and the wave number acquires an imaginary part: *k* → *k′* = *k* − *i*ln *r*. The legitimate values of *β* trace out a trajectory in the complex plane, known as the generalized Brillouin zone (GBZ). The corresponding GBZ Hamiltonian is given by:


(5) \begin{eqnarray*}
{{H}}\left( \beta \right) = \left( { - i} \right)\left[ {\begin{array}{@{}*{2}{c}@{}} {{P}_2 - {P}_3 + {P}_1\beta - {P}_1{\beta }^{ - 1}}&{{P}_3 - {P}_2\beta }\\ { - {P}_3 + {P}_2{\beta }^{ - 1}}&{{P}_3 - {P}_2} \end{array}} \right].
\end{eqnarray*}

The precise shape of the GBZ can be found as follows. From the eigenvalue function det[*H*(*β*) *−* *EI*] = 0, a given *E* corresponds to two *β* roots denoted as *β*_1_(*E*) and *β*_2_(*E*), then the solution of the equation *β*_1_(*E*) = *β*_2_(*E*) determines the legitimate values of *E* and *β*. These values of *β* form a continuous contour in the complex plane that is the GBZ and the corresponding eigenvalues *E* are obtained to analyse the band structure and topological properties of the RPS chain.

**Figure 4.**
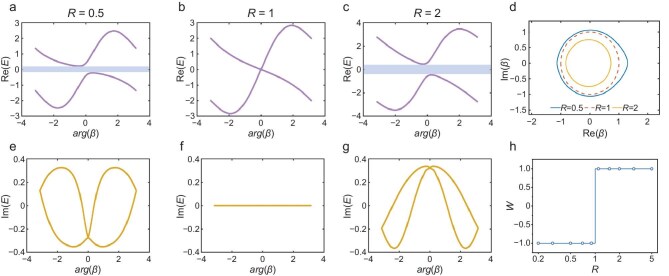
Band structure of thermal RPS Hamiltonian in GBZ. (a–c) Real part of eigenvalues of *H*(*k*) with skewness *R* = 0.5, 1 and 2. (d) GBZ of *H*(*k*) with skewness *R* = 0.5, 1 and 2. The dashed line is the unit circle (BZ, when *R* = 1). (e–g) Imaginary part of eigenvalues of *H*(*k*) with skewness *R* = 0.5, 1 and 2. (h) Topological invariant *W* of thermal RPS chain with different skewness *R*.

Figure 4d illustrates the GBZ with skewness *R* = 0.5, 1 and 2. For skewness *R* = 1, the GBZ is degenerated into a BZ, which is a unit circle. For *R* < 1, the GBZ constitutes a continuous contour lying entirely outside the unit circle. Conversely, for *R* > 1, the contour lies entirely inside the unit circle. In all cases, the GBZ is not circular, but a continuous curve symmetric about the real axis [Im(*β*) = 0] except for *R* = 1. The band structures of the thermal RPS chain in the GBZ are depicted in Fig. 4a–c and e–g, in which the horizontal axis is the argument *arg*(*β*). Here, *arg*(*β*) = Re(*k′*), which represents the real part of the complex wavevector in the GBZ framework. The real part Re(*E*) (Fig. 4a–c) and imaginary part Im(*E*) (Fig. 4e–g) of the eigenvalue of *H*(*β*) represent the transmission and the dissipation of thermal energy, respectively. First, the spectrum of Re(*E*) exhibits two bands of eigenvalues on the GBZ, reflecting the two sides A and B of a unit. For *R* = 1, the two branches intersect at *arg*(*β*) = 0, whereas, for *R* ≠ 1, they are separated by a spectral gap. This gap closes only for *R* = 1 at *arg*(*β*) = 0, supporting the view that *R* = 1 is the only critical point for topological phase transition. Secondly, a clear transition is also observed in Im(*E*). For *R* = 1, *H*(*β*) is Hermitian and all eigenvalues are pure real, denoting the thermal dissipative behavior only. In contrast, for *R* ≠ 1, Im(*E*) presents a distribution with axial symmetry and the band branches intersect at *arg*(*β*) = 0. This marked change in the imaginary band structure across the critical point *R* = 1 further corroborates the occurrence of a phase transition.

Since the eigenenergy spectrum collapses into an arc in the GBZ (a detailed comparison between the BZ and the GBZ is shown in Note SIII), the topological invariant (winding number *W*) is calculated in the BZ as:


(6) \begin{eqnarray*}
W = \frac{1}{{2\pi }}\oint_{BZ} {\frac{d}{{dk}}\arg \left[ {H( k) - {E}_{\rm b}} \right]} dk,
\end{eqnarray*}

where *E*_b_ is the reference point. Essentially, *W* gives the number of times the complex eigenenergy encircles *E*_b_. This topological invariant *W* serves as an indicator of the topological phase of the thermal RPS chain. The relationship between *W* and the skewness *R* are shown in Fig. 4h. *W* = −1 and +1 correspond to temperature localization toward the right (*R* < 1) and left (*R* > 1), respectively. A topological phase transition occurs at *R* = 1 and these two phases (*R* < 1 and *R* > 1) are topologically distinct, as one cannot be deformed into the other without crossing the critical point.

**Figure 5.**
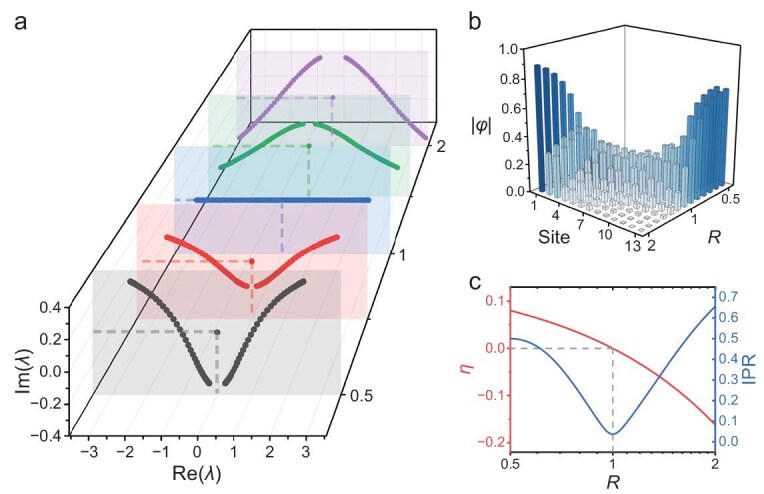
Eigenvalues and eigenvectors of finite thermal RPS chain under OBC. (a) Eigenvalues of real space Hamiltonian with different skewness (*s* = 81). (b) Eigenvectors corresponding to zero eigenvalues with different skewness (*s* = 13). (c) Normalized temperature deviation *η* and IPR under steady-state conditions with skewness varying from 0.5 to 2.

The eigenvalues *λ* and eigenvectors of the OBC Hamiltonian for a finite thermal RPS chain provide further evidence of the phase transition. The Hamiltonian follows the form given in Supplementary Equation S9 and the results are illustrated in Fig. 5a (*s* = 81). As *R* increases across the critical value *R* = 1, the two spectral branches exhibit a reversal in their opening direction. The branch of *R* < 1 opens upward whereas the branch of *R* > 1 opens downward. Moreover, an obvious gap emerges at *λ* = 0 if *R* ≠ 1. This zero point corresponds to an eigenmode in which both propagation [Re(*λ*) = 0] and dissipation [Im(*λ*) = 0] cease, indicating the steady state of the thermal RPS chain. The associated eigenvector is thus referred to as the steady-state eigenvector. Further investigation of the steady-state eigenvector is presented in Fig. 5b (*s* = 13). As for *R* < 1, the steady-state eigenvectors localize toward the left (the region with the smaller *N*), with the degree of localization becoming more pronounced as *R* decreases. Conversely for *R* > 1, the localization occurs toward the right (larger *N*), strengthening as *R* increases. At *R* = 1, the eigenvectors are uniformly distributed across all sites. This spatial localization behavior aligns fully with the observed temperature localization patterns. It is worth noting that this localization is different from the reported thermal skin effect [29,30], which focuses more on the temperature distribution/eigenvectors during transient processes whereas we are more concerned with the steady state. This is because the reported thermal skin effect is a purely passive dissipation process—under adiabatic boundary conditions, heat will inevitably evolve toward a uniform distribution—whereas, in our model, due to the active thermal control driven by thermoelectric materials, the temperature field evolves from an initial distribution and continuously accumulates toward a certain direction, eventually forming a stable temperature localization profile. To quantitatively characterize this localization, we introduce the inverse participation ratio (IPR), defined as $IPR = {\sum {| {{\phi }_i} |} }^4$, where ${\phi }_i$ denotes the amplitude at site *i*. A large IPR usually corresponds to localization. Figure 5c shows the normalized temperature deviation and IPR under steady-state conditions with *R* varying from 0.5 to 2. At *R* = 1, the normalized temperature deviation changes sign and the IPR reaches its minimum. These two indicators jointly show that the localization of temperature and the localization of the steady-state eigenvector are highly consistent in both direction and magnitude. The agreement between topological band theory and actual heat-transport behavior strongly supports the existence of a topological phase transition in the thermal RPS chain.

## DISCUSSION AND CONCLUSION

Inspired by the cyclic restraint relationships between biological populations in nature, we establish a thermal RPS chain and investigate the robust temperature localization and topological phase transition by using both phenomenology and topological band-theory analysis. The influence of the skewness parameter *R* on the direction of temperature localization is analysed, as well as the robustness of the thermal RPS chain under parameter and structural perturbations. Further, the topological phase transition that occurs at *R* = 1 in the thermal RPS chain is clarified through both the temperature localization phenomena and band-structure analysis. Owing to the progress in high-efficiency thermoelectric materials and 3D printing, the ability of the thermal RPS chain to robustly steer and localize heat to a designated boundary on demand has become possible. This work successfully establishes a non-equilibrium thermal transport model at the macroscopic level, laying the foundation for more topological thermal transfer phenomena and paving the way for potential advanced thermal management technologies.

## METHODS

The robust topological temperature localization in the thermal RPS chain is verified by using the finite element simulations. In the simulation, we construct an OBC model that consists of 13 sites (*N* = 6), as shown in Fig. 2. The distance between each site is 30 mm. The material is set as copper with thermal conductivity *κ* = 400 W· m^−1^· K^−1^, density *ρ* = 8.9 × 10^3^ kg· m^−3^ and capacity *c* = 390 J· kg^−1^· K^−1^. The initial temperature is set as 293.15 K and the boundary condition is set as adiabatic. The simulated results are shown in Figs 2 and 3.

## Supplementary Material

nwag169_Supplemental_File

## References

[1] Li M, Zhirihin D, Gorlach M et al. Higher-order topological states in photonic kagome crystals with long-range interactions. Nat Photon 2020; 14: 89–94. 10.1038/s41566-019-0561-9

[2] Benalcazar WA, Bernevig BA, Hughes TL. Quantized electric multipole insulators. Science 2017; 357: 61–6. 10.1126/science.aah6442 28684520

[3] Guo J, Gu Z, Zhu J. Realization of merged topological corner states in the continuum in acoustic crystals. Phys Rev Lett 2024; 133: 236603. 10.1103/PhysRevLett.133.236603 39714643

[4] Fleury R, Sounas DL, Sieck CF et al. Sound isolation and giant linear nonreciprocity in a compact acoustic circulator. Science 2014; 343: 516–9. 10.1126/science.1246957 24482477

[5] Zhang X, Zhang T, Lu M-H et al. A review on non-Hermitian skin effect. Adv Phys-X 2022; 7: 2109431. 10.1080/23746149.2022.2109431

[6] El-Ganainy R, Makris KG, Khajavikhan M et al. Non-Hermitian physics and PT symmetry. Nat Phys 2018; 14: 11–9. 10.1038/nphys4323

[7] Weidemann S, Kremer M, Helbig T et al. Topological funneling of light. Science 2020; 368: 311–4. 10.1126/science.aaz8727 32217752

[8] Liu Z, Jin P, Lei M et al. Topology in thermal, particle, and plasma diffusion metamaterials. Chem Rev 2025; 125: 8655–730. 10.1021/acs.chemrev.4c00912 40920975 PMC12494037

[9] Jin P, Wang C, Zhou Y et al. Temporal anti-parity–time symmetry in diffusive transport. Nat Phys 2026; 22: 195–201. 10.1038/s41567-025-03129-8

[10] Zhuang P, Wang C, Yang F et al. Rescaled Schwarz–Christoffel transformations for isotropic, polygon, and multiphysics metamaterials. Phys Rev Lett 2025; 135: 216901. 10.1103/nzvh-lxr8 41349065

[11] Fan CZ, Gao Y, Huang JP. Shaped graded materials with an apparent negative thermal conductivity. Appl Phys Lett 2008; 92: 251907. 10.1063/1.2951600

[12] Liu Q, Wang Z, Kim S-K et al. Remote spatiotemporal control of local states in thermal lattice. Mater Today Phys 2025; 59: 101954. 10.1016/j.mtphys.2025.101954

[13] Wang Z, Liu Q, Xiang L et al. Macroscale anomalous heat conduction in active thermal metamaterials. Newton 2025; 1: 100255. 10.1016/j.newton.2025.100255

[14] Li Y, Xu L, Qiu C-W. Thermal Metamaterials: Controlling the Flow of Heat. Singapore: World Scientific, 2025. 10.1142/13874

[15] Yang F, Zhang Z, Xu L et al. Controlling mass and energy diffusion with metamaterials. Rev Mod Phys 2024; 96: 015002. 10.1103/RevModPhys.96.015002

[16] Sha W, Xiao M, Zhang J et al. Robustly printable freeform thermal metamaterials. Nat Commun 2021; 12: 7228. 10.1038/s41467-021-27543-7 34893631 PMC8664938

[17] Wang Z, Zhu Z, Liu T et al. Inverse design of thermal metamaterials with holey engineering strategy. J Appl Phys 2022; 132: 145102. 10.1063/5.0108743

[18] Zhu Z, Wang Z, Liu T et al. Arbitrary-shape transformation multiphysics cloak by topology optimization. Int J Heat Mass Transfer 2024; 222: 125205. 10.1016/j.ijheatmasstransfer.2024.125205

[19] Zhu Z, Ren X, Sha W et al. Inverse design of rotating metadevice for adaptive thermal cloaking. Int J Heat Mass Transfer 2021; 176: 121417. 10.1016/j.ijheatmasstransfer.2021.121417

[20] Sha W, Hu R, Xiao M et al. Topology-optimized thermal metamaterials traversing full-parameter anisotropic space. Npj Comput Mater 2022; 8: 179. 10.1038/s41524-022-00861-0

[21] Xu H, Shi X, Gao F et al. Ultrathin three-dimensional thermal cloak. Phys Rev Lett 2014; 112: 054301. 10.1103/PhysRevLett.112.054301 24580599

[22] Hu R, Xi W, Liu Y et al. Thermal camouflaging metamaterials. Mater Today 2021; 45: 120–41. 10.1016/j.mattod.2020.11.013

[23] Narayana S, Sato Y. Heat flux manipulation with engineered thermal materials. Phys Rev Lett 2012; 108: 214303. 10.1103/PhysRevLett.108.214303 23003263

[24] Hu R, Zhou S, Li Y et al. Illusion thermotics. Adv Mater 2018; 30: 1707237. 10.1002/adma.201707237 29665110

[25] Hu R, Huang S, Wang M et al. Encrypted thermal printing with regionalization transformation. Adv Mater 2019; 31: 1807849. 10.1002/adma.201807849 31058371

[26] Li J, Li Y, Cao P-C et al. Reciprocity of thermal diffusion in time-modulated systems. Nat Commun 2022; 13: 167. 10.1038/s41467-021-27903-3 35013296 PMC8748696

[27] Xu L, Xu G, Huang J et al. Diffusive Fizeau drag in spatiotemporal thermal metamaterials. Phys Rev Lett 2022; 128: 145901. 10.1103/PhysRevLett.128.145901 35476493

[28] Ju R, Cao P, Wang D et al. Nonreciprocal heat circulation metadevices. Adv Mater 2024; 36: 2309835. 10.1002/adma.202309835 38010625

[29] Liu Y-K, Cao P-C, Qi M et al. Observation of non-Hermitian skin effect in thermal diffusion. Sci Bull 2024; 69: 1228–36. 10.1016/j.scib.2024.02.040 38503653

[30] Cao P-C, Li Y, Peng Y-G et al. Diffusive skin effect and topological heat funneling. Commun Phys 2021; 4: 230. 10.1038/s42005-021-00731-z

[31] Qi M, Wang D, Cao P et al. Geometric phase and localized heat diffusion. Adv Mater 2022; 34: 2202241. 10.1002/adma.202202241 35676890

[32] Xu G, Li Y, Li W et al. Configurable phase transitions in a topological thermal material. Phys Rev Lett 2021; 127: 105901. 10.1103/PhysRevLett.127.105901 34533332

[33] Xu G, Yang Y, Zhou X et al. Diffusive topological transport in spatiotemporal thermal lattices. Nat Phys 2022; 18: 450–6. 10.1038/s41567-021-01493-9

[34] Liu Z, Cao P-C, Xu L et al. Higher-order topological in-bulk corner state in pure diffusion systems. Phys Rev Lett 2024; 132: 176302. 10.1103/PhysRevLett.132.176302 38728705

[35] Wang Z, Liu T, Zhu Z et al. Periodicity alters topological states in thermal diffusion system. Int J Heat Mass Transfer 2024; 235: 126182. 10.1016/j.ijheatmasstransfer.2024.126182

[36] Wu H, Hu H, Wang X et al. Higher-order topological states in thermal diffusion. Adv Mater 2023; 35: 2210825. 10.1002/adma.202210825 36730361

[37] Hu H, Han S, Yang Y et al. Observation of topological edge states in thermal diffusion. Adv Mater 2022; 34: 2202257. 10.1002/adma.202202257 35674403

[38] Yoshida T, Hatsugai Y. Bulk-edge correspondence of classical diffusion phenomena. Sci Rep 2021; 11: 888. 10.1038/s41598-020-80180-w 33441795 PMC7806654

[39] Liu F, Wakabayashi K. Novel topological phase with a zero Berry curvature. Phys Rev Lett 2017; 118: 076803. 10.1103/PhysRevLett.118.076803 28256872

[40] Shankar S, Souslov A, Bowick MJ et al. Topological active matter. Nat Rev Phys 2022; 4: 380–98. 10.1038/s42254-022-00445-3

[41] Fruchart M, Hanai R, Littlewood PB et al. Non-reciprocal phase transitions. Nature 2021; 592: 363–9. 10.1038/s41586-021-03375-9 33854249

[42] Zhao S, Tian Z, Shen C et al. Topological acoustofluidics. Nat Mater 2025; 24: 707–15. 10.1038/s41563-025-02169-y 40119033 PMC12048345

[43] Liu Q, Wang Z, Zeng M et al. Dynamically adjustable topological edge states in thermal diffusion-advection system. Fundam Res 2025; doi: 10.1016/j.fmre.2025.02.001 10.1016/j.fmre.2025.02.001. PMC13424978 42540056

[44] Knebel J, Geiger PM, Frey E. Topological phase transition in coupled rock-paper-scissors cycles. Phys Rev Lett 2020; 125: 258301. 10.1103/PhysRevLett.125.258301 33416395

[45] Yoshida T, Mizoguchi T, Hatsugai Y. Chiral edge modes in evolutionary game theory: a kagome network of rock-paper-scissors cycles. Phys Rev E 2021; 104: 025003. 10.1103/PhysRevE.104.025003 34525642

[46] Felski A, Kunst FK. Exceptional points and stability in nonlinear models of population dynamics having PT symmetry. Phys Rev Res 2025; 7: 013326. 10.1103/PhysRevResearch.7.013326

[47] Liang J, Dai Q, Li H et al. Topological phases in population dynamics with rock-paper-scissors interactions. Phys Rev E 2024; 110: 034208. 10.1103/PhysRevE.110.034208 39425366

[48] Kerr B, Riley MA, Feldman MW et al. Local dispersal promotes biodiversity in a real-life game of rock–paper–scissors. Nature 2002; 418: 171–4. 10.1038/nature00823 12110887

[49] Kirkup BC, Riley MA. Antibiotic-mediated antagonism leads to a bacterial game of rock–paper–scissors in vivo. Nature 2004; 428: 412–4. 10.1038/nature02429 15042087

[50] Buss LW, Jackson JBC. Competitive networks: nontransitive competitive relationships in cryptic coral reef environments. Am Nat 1979; 113: 223–34. 10.1086/283381

[51] Sinervo B, Lively CM. The rock–paper–scissors game and the evolution of alternative male strategies. Nature 1996; 380: 240–3. 10.1038/380240a0

[52] Zhang K, Yang Z, Fang C. Correspondence between winding numbers and skin modes in non-Hermitian systems. Phys Rev Lett 2020; 125: 126402. 10.1103/PhysRevLett.125.126402 33016766

