# Supplementary material for: Robust topological temperature localization in thermal rock–paper–scissors chain
Source: Natl Sci Rev. 2026 Mar 20;13(11):nwag169. doi: 10.1093/nsr/nwag169 (PMC13224842; doi:10.1093/nsr/nwag169)
Supplement: nwag169_Supplemental_File [file nwag169_supplemental_file.docx]

## **Supplementary Data for**

## **“Robust Topological Temperature Localization in Thermal Rock-Paper-Scissors Chain”**

Zhaochen Wang^1^, Quan Liu^1^, Xin Qian^1^, Xiaobing Luo^1^, Run Hu^1,2,3,4,*^

^1^School of Energy and Power Engineering, Huazhong University of Science and Technology, Wuhan 430074, China

^2^State Key Laboratory of High-Density Electrical Energy Conversion, Huazhong University of Science and Technology, Wuhan 430074, China

^3^Department of Applied Physics, Kyung Hee University, Yongin-Si 17104, Korea

^4^Shenzhen Institute of Huazhong University of Science and Technology, Shenzhen 518052, China

^*^Corresponding author. E-mail: [hurun@hust.edu.cn](mailto:hurun@hust.edu.cn)

# **Supplemental Note I: Derivation of Thermal RPS Chain**

In supplemental note I, we show the setup of Peltier module and the derivation procedure of the discretized thermal diffusion equation. Without loss of generality, we consider an active thermal structure with non-uniformly distributed active transport and passive diffusion (Fig. S1).


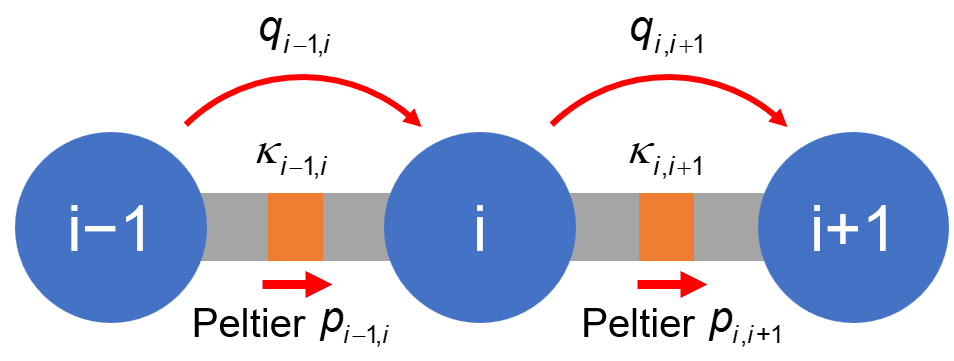


FIG. S1. Schematic of active thermal structure consisting of sites connected with Peltier channels.

With Peltier module set at each channel, heat will be absorbed at one site and generate at the other site, thus heat can be transport actively through the channel between these two sites, forming an antisymmetric transport coefficient. We assume that this ideal Peltier module has sufficiently low resistance. Additionally, since it is positioned at the center of the channel, its temperature is regarded as the average temperature of the two sites. heat flow through the Peltier module is at a rate of *Q* = *SIT*_avg_, where *S*, *I*, and *T*_avg_ are the Seebeck coefficient, the electric current, and the average temperature of the channel.

With the assumption that the temperature is almost uniform within each site, the temperature of each site is denoted as *T_i_* with subscript corresponding to the site index. The temperature of each site is influenced by the energy change between channels connected with other sites. According to Fourier’s law, the governing equation of *T_i_* is

$\rho c\frac{\partial T_{i}}{\partial t}=-\nabla q=\frac{q_{i-1,i}-q_{i,i+1}}{a_{0}}$ (S1)

where *q_i−1,i_* (*q_i,i+1_*) is the heat flow in channel *i−1,i* (channel *i,i+1*), *ρ* and *c* are the density and heat capacity of the structure, *a_0_* is the distance between adjacent two sites. The discretized heat flow is

$\left\{\begin{matrix} q_{i-1,i}=\kappa_{i-1,i}\frac{T_{i-1}-T_{i}}{a_{0}}+SI_{i-1,i}\frac{T_{i-1}+T_{i}}{2A_{0}} \\ q_{i,i+1}=\kappa_{i,i+1}\frac{T_{i}-T_{i+1}}{a_{0}}+SI_{i,i+1}\frac{T_{i}+T_{i+1}}{2A_{0}} \end{matrix}\right.$ (S2)

where *κ_i−1,i_* (*κ_i,i+1_*)and *I_i−1,i_* (*I_i,i+1_*) are the thermal conductivity and the current of the channel *i−1,i* (channel *i,i+1*), *A_0_* is the cross-section area of the channel. Then the Eq. (S1) is

$\frac{\partial T_{i}}{\partial t}=\frac{1}{\rho ca_{0}}\left[\left(\frac{SI_{i-1,i}}{2A_{0}}+\frac{\kappa_{i-1,i}}{a_{0}}\right)T_{i-1}+\left(-\frac{SI_{i,i+1}}{2A_{0}}+\frac{\kappa_{i,i+1}}{a_{0}}\right)T_{i+1}+\left(\frac{SI_{i-1,i}}{2A_{0}}-\frac{SI_{i,i+1}}{2A_{0}}-\frac{\kappa_{i-1,i}}{a_{0}}-\frac{\kappa_{i,i+1}}{a_{0}}\right)T_{i}\right]$(S3)

Set the active transport coefficient *P_i,i+1_* and the diffusion coefficient *D_i,i+1_* to characterize the heat exchange resulting from active driving and passive diffusion:

$\left\{\begin{matrix} P_{i,i+1}=\frac{SI_{i,i+1}}{2\rho ca_{0}A_{0}} \\ D_{i,i+1}=\frac{\kappa_{i,i+1}}{\rho ca_{0}^{2}} \end{matrix}\right.$ (S4)

Then the Eq. (S3) can be written as

$\partial_{t}T_{i}=(P_{i-1,i}+D_{i-1,i})T_{i-1}+(-P_{i,i+1}+D_{i,i+1})T_{i+1}+(P_{i-1,i}-P_{i,i+1}-D_{i-1,i}-D_{i,i+1})T_{i}$ (S5)

Such a discrete heat transfer model represents the temperature of each site as an expression involving the temperatures of adjacent site and two coefficients (*P_i,i+1_* and *D_i,i+1_*).

With this model, we represent the temperature evolution of thermal RPS chain as shown in Fig.1 as an expression involving site temperatures, active transport coefficients *P_j_*, and diffusion coefficients *D_j_* (*j* = 1, 2, 3) as

$\left\{\begin{matrix} \begin{matrix} \frac{\partial T_{n,A}}{\partial t}=D_{1}\left(T_{n+1,A}-T_{n,A}\right)+D_{1}\left(T_{n-1,A}-T_{n,A}\right) \\ +D_{2}\left(T_{n-1,B}-T_{n,A}\right)+D_{3}\left(T_{n,B}-T_{n,A}\right) \\ +P_{3}\left(T_{n,B}+T_{n,A}\right)+P_{1}\left(T_{n-1,A}+T_{n,A}\right) \\ -P_{2}\left(T_{n-1,B}\text{+}T_{n,A}\right)-P_{1}\left(T_{n+1,A}+T_{n,A}\right) \end{matrix} \\ \begin{matrix} \frac{\partial T_{n,B}}{\partial t}=D_{2}\left(T_{n+1,A}-T_{n,B}\right)+D_{3}\left(T_{n,A}-T_{n,B}\right) \\ +P_{2}\left(T_{n+1,A}+T_{n,B}\right)-P_{3}\left(T_{n,A}+T_{n,B}\right) \end{matrix} \end{matrix}\right.$ (S6)

where *T_n,A_* and *T_n,B_* denote the temperature of site A and B in unit *n*. The linear equations can be written into a single matrix equation as

$\frac{\partial\hat{\mathbf{T}}}{\partial t}=-i\mathbf{H}\hat{\mathbf{T}}$ (S7)

where $\hat{\mathbf{T}}=\left[\begin{matrix} T_{1,A} & T_{1,B} & \cdots& T_{n,B} \end{matrix}\right]^{T}$ represents the temperature field of whole RPS chain, **H** corresponds to the Hamiltonian operator and *i* is the imaginary unit. For theoretical analysis with periodic boundary conditions (PBC), we close the chain by coupling the last B site back to the first A site, forming a ring with an even total number of sites *s* = 2*N*, where *N* is the total number of units. For open boundary conditions (OBC) used in most situations, the chain is terminated, resulting in an odd total number of sites *s* = 2*N*+1. Consider an OBC thermal RPS chain, the corresponding Hamiltonian can be written as

$\text{H}=\left(-i\right)\cdot\left[\begin{matrix} D_{1}+D_{3}+P_{1}-P_{3} & -D_{3}-P_{3} & -D_{1}+P_{1} & \cdots& & & \\ -D_{3}+P_{3} & D_{2}+D_{3}+P_{3}-P_{2} & -D_{2}-P_{2} & \cdots& & & \\ -D_{1}-P_{1} & -D_{2}+P_{2} & 2D_{1}+D_{2}+D_{3}+P_{2}-P_{3} & -D_{3}-P_{3} & -D_{1}+P_{1} & \cdots& \\ \vdots& \vdots& -D_{3}+P_{3} & D_{2}+D_{3}+P_{3}-P_{2} & -D_{2}-P_{2} & \cdots& \\ & & \vdots& \vdots& \ddots& \vdots& \vdots\\ & & & \cdots& -D_{3}+P_{3} & D_{2}+D_{3}+P_{3}-P_{_{2}} & -D_{2}-P_{2} \\ & & & \cdots& -D_{1}-P_{1} & -D_{2}+P_{2} & D_{1}+D_{2}+P_{2}-P_{1} \end{matrix}\right]$(S8)

When *P_j_* >> *D_j_* (*j* = 1,2,3), thus *D_j_* in the Hamiltonian can be neglected, and the Hamiltonian **H** becomes antisymmetric as

$H=\left(-i\right)\cdot\left[\begin{matrix} P_{1}-P_{3} & -P_{3} & P_{1} & \cdots& & & \\ P_{3} & P_{3}-P_{2} & -P_{2} & \cdots& & & \\ -P_{1} & P_{2} & P_{2}-P_{3} & -P_{3} & P_{1} & \cdots& \\ \vdots& \vdots& P_{3} & P_{3}-P_{2} & -P_{2} & \cdots& \\ & & \vdots& \vdots& \ddots& \vdots& \vdots\\ & & & \cdots& P_{3} & P_{3}-P_{_{2}} & -P_{2} \\ & & & \cdots& -P_{1} & P_{2} & P_{2}-P_{1} \end{matrix}\right]$ (S9)

# **Supplemental Note Ⅱ: Temperature-Weighted Geometric Center of Thermal RPS Chain**

To quantitatively describe the polarization of thermal RPS chain, we introduce the temperature-weighted geometric center as

$\overline{x}_{T}=\frac{\int_{0}^{L} xT\left(x\right)dx}{\int_{0}^{L} T\left(x\right)dx}=\frac{\sum_{1}^{s} i\cdot T_{i}}{\sum_{1}^{s} T_{i}}a_{0}$ (S10)

where *x* is the position, *T*(*x*) is the corresponding temperature, *L* is the total length of thermal RPS chain, *s* is the total number of sites, *a_0_* is the distance between adjacent sites. At the same time, we consider the geometric center of the thermal RPS chain as

$\overline{x}_{g}=\frac{L}{2}=\frac{s+1}{2}a_{0}$ (S11)

For a given thermal RPS chain, since the geometric structure remains unchanged, the geometric center will not change. However, when temperature localization occurs, the degree of temperature deviation will cause the temperature distribution center to change. Therefore, by examining the deviation between the geometric center and the temperature distribution center, the degree of temperature polarization can be evaluated. We proposed the normalized temperature deviation *η* as

$\eta=\frac{\bar{x}_{T}-\bar{x}_{g}}{a_{0}}=\frac{\sum_{1}^{s} i\cdot T_{i}}{\sum_{1}^{s} T_{i}}-\frac{s+1}{2}$ (S12)

For steady state, the relationship between *η* and *R* is illustrated in Fig. S2(a). When *R* = 1, *η* = 0 and the temperature distribution center coincides with the geometric center. When *R* < 1, *η* > 0 and increases with the decrease of *R*, indicating that the overall temperature undergoes polarization to the right. When *R* > 1, *η* < 0 and decreases with the increase of *R*. Now the shift direction is opposite. Both for *R* < 1 and *R* > 1, the greater the absolute value of *η*, the greater the polarization strength will be.

Fig. S2(a) illustrates the relationship between *η* and R under different disturbance situations. The red line shows the results without disturbance. The blue dots show the results when applying disturbance on active transport coefficient. The gray dots show the results when applying structural perturbations and extra couplings. Coefficient disturbance will randomly increasing or decreasing *η* because this disturbance is drived by a random change on the active transport coefficient. As for structural perturbations, *η* will always decrease because the applied extra couplings have the uniform direction (towards the left). These extra couplings make the temperature field undergo polarization to the left which corresponds to smaller *η*.

For transient state, the relationship between *η* and time *t* is illustrated in Fig. S2(b). The value of *η* increases with time, and its sign depends on the value of *R*. When *R* < 1, *η* > 0 while when *R* > 1, *η* < 0 [Fig. S2(b)].


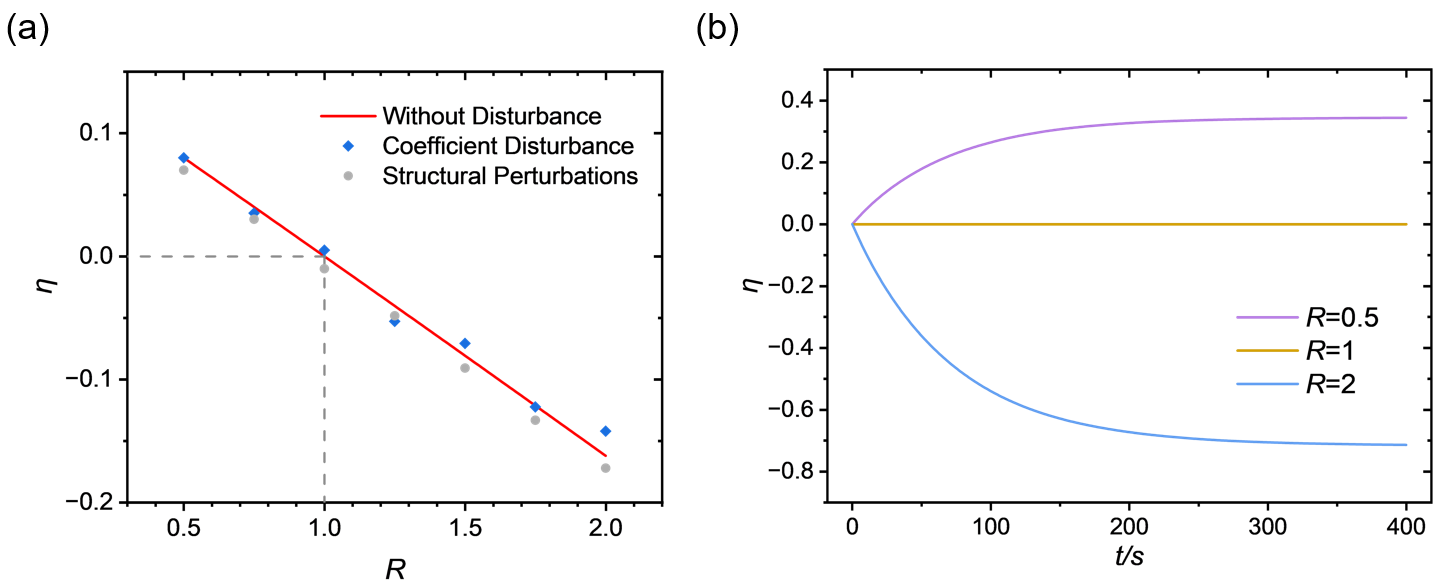


FIG. S2. Normalized temperature deviation of thermal RPS chain. (*S* = 13) (a) Steady state normalized temperature deviation with skewness varying from 0.5 to 2 with consistent *P*_1_. The red line refers to results without disturbance. The blue dots refer to results with coefficient disturbance and the gray dots refers to results with structural perturbations (b) Transient state normalized temperature deviation when *R* = 0.5, 1 and 2 with consistent *P*_1_.

# **Supplemental Note Ⅲ: Comparison of BZ and GBZ**

In contrast to the BZ with the phase factor *e^ik^* traces a unit circle as *k* winds from −*π* to *π*, the GBZ with the generalized phase factor *β* = *re^ik^* generally deviates from the unit circle as *r* typically differs from 1. This results in the difference between the energy spectra on BZ and GBZ [Figs. S3(a-c)]. For skewness *R*=1, GBZ is degenerated into BZ and their energy spectra coincide. In this case, although thermal energy transfers between sites, the system reaches a dynamic equilibrium with a uniform temperature distribution that remains constant over time. Under this condition, PBC and OBC yield identical system characteristics, and the eigenvalue distributions in real space are also similar [Figs. S3(e)]. For skewness *R* ≠ 1, there will be significant differences between the BZ and GBZ spectra. The BZ spectrum always forms closed loops, enabling the winding number *W* defined in Eq. (6) to infer the topological state of the system. In contrast, the eigenenergy on GBZ no longer forms any loops nor encircles any base points, but instead collapses to arcs. Accordingly, the winding number on GBZ is either zero or ill-defined. Nevertheless, the well-defined, nonzero *W* computed on BZ remains a valid indicator.

This spectral correspondence can be extended to real space: PBC results align with the BZ description, while OBC results correspond to the GBZ, as illustrated in Figs. S3(d)–(f). The OBC spectrum collapses from a loop on the complex plane into an arc. These findings demonstrate that the GBZ effectively restores the bulk-edge correspondence in *k*-space and captures topological features under OBC. Beyond predicting the OBC spectrum, the GBZ also encodes information about the corresponding eigenstates. Specifically, any part of the GBZ that lies within (without) the unit circle corresponds to a set of skin modes. In other words, any deviation of the GBZ from the BZ (when *R* ≠ 1) signals the emergence of temperature localization.


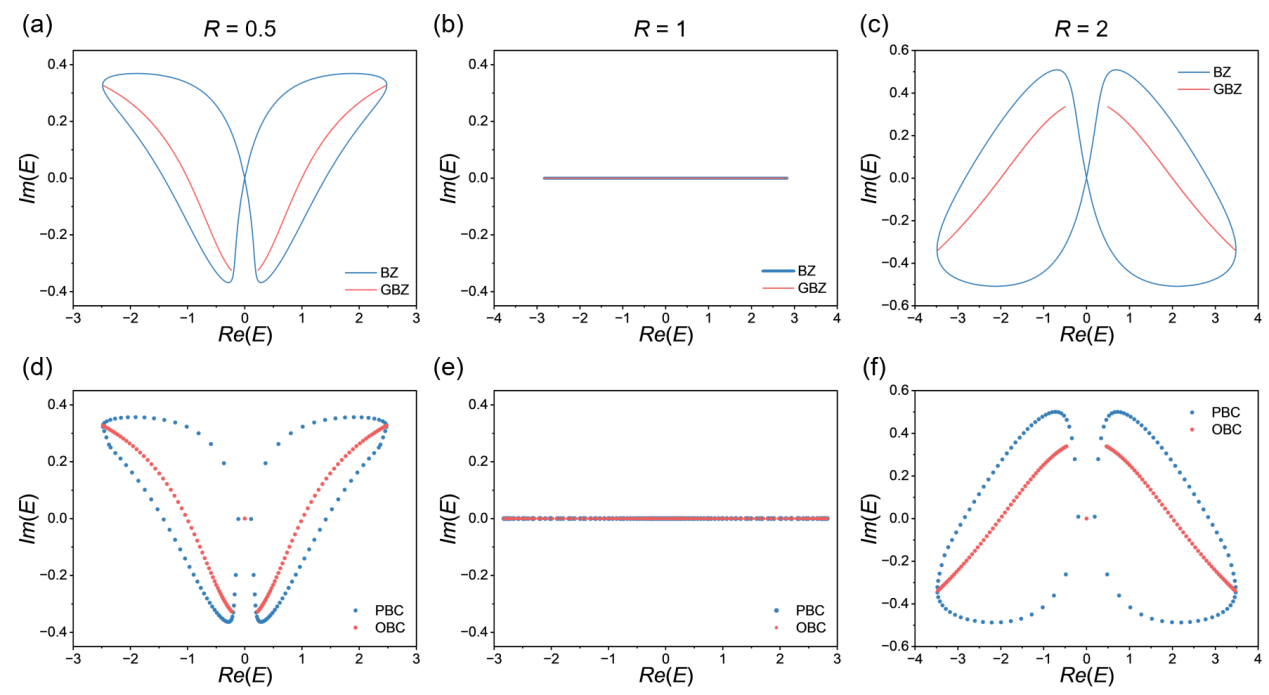


FIG. S3. Spectrum of thermal RPS chain. (a-c) Energies in BZ (blue) and GBZ (red). (d-f) Eigenvalues of real-space Hamiliton of finite thermal RPS chain under PBC (S = 120) and OBC (S = 121).

# **Supplemental Note Ⅳ: Eigenmodes in Real Space under Different Boundary Conditions and Different Values of *P*_1_**

Boundary conditions have a crucial influence on the topological phenomena of non-Hermitian systems. To better illustrate the topological temperature localization phenomena, we consider three types of boundary conditions: open boundary conditions (OBC), periodic boundary conditions (PBC), and left-boundary (LB). Consider a thermal RPS chain with *N* unit cells. Under OBC [Fig. S4(a)], we terminate the chain by adding an extra A site after the *N*th unit cell to ensure that the last unit also forms a complete RPS cycle. Then the chain consists of *s* = 2*N*+1 sites. Under PBC [Fig. S4(b)], the chain forms a ring with s = 2*N*, where the last unit cell couples back to the first A site (site 1). As to LB thermal RPS chain [Fig. S4(c)], the last unit cell is not connected to site 1 and there is also no extra site. This system has the same left boundary as OBC (thus, “LB” as subscript), but a different right boundary.

The results of the steady-state eigenvectors are shown in Figs. S4(d-l). Under PBC, the localization phenomenon no longer occurs since there are no boundaries. This conclusion is consistent with other studies on the non-Hermitian skin effect. As long as the thermal RPS chain is terminated with a clear boundary, the localization will occur whether under OBC or LB. Since the left boundary is the same, when the temperature localizes to the left (*R* > 1), the distribution of steady-state eigenvectors shows extremely high similarity under OBC and LB. At this time, the contributions from the right boundary are almost non-existent in the eigenvectors. While when temperature localizes to the right (*R* < 1), the inconsistency in the structure of the right boundary is clearly manifested in the distribution of eigenvectors. The eigenvectors under OBC are concentrated on the two rightmost sites, while under LB case they are distributed on the three rightmost sites.


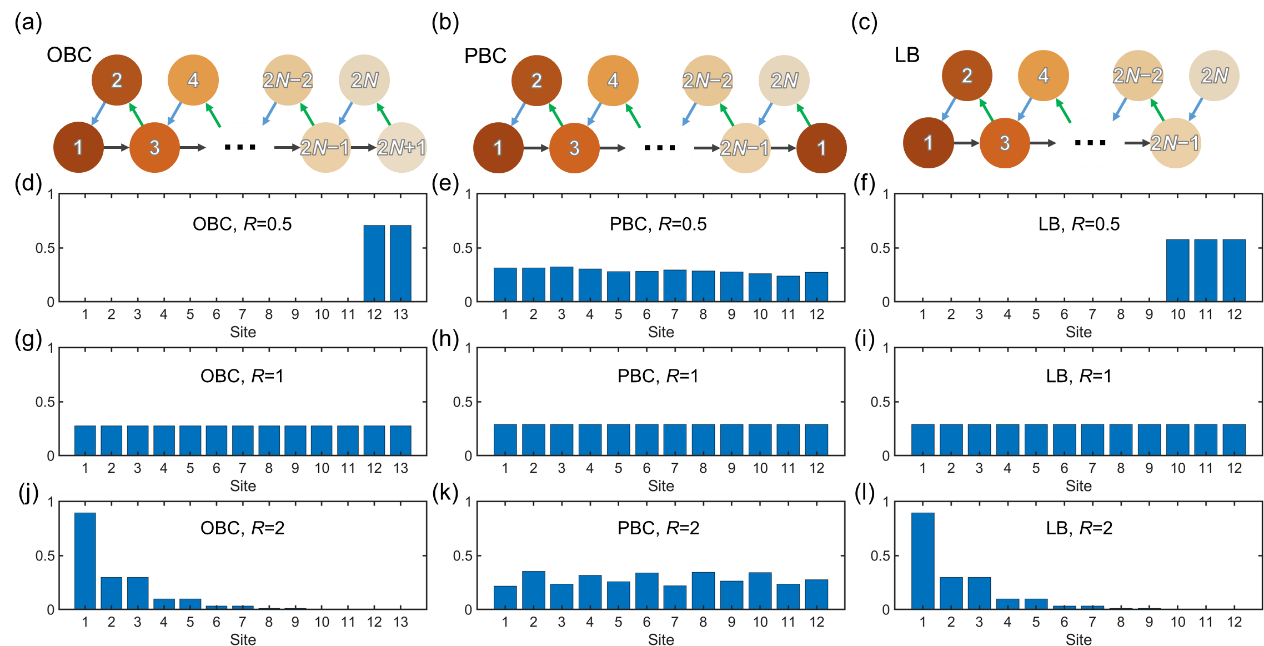


FIG. S4. Steady-state eigenvectors of thermal RPS chain under different boundary conditions. (a-c) Schematic of three different boundary conditions. (d-l) Steady-state eigenvectors (*N* = 6) under different boundary conditions with skewness *R* = 0.5 (d-f), 1 (g-i), 2 (j-l).

This work mainly focuses on the interrelationship between *P*_2_ and *P*_3_, that is, the changes in the value of skewness *R* and the corresponding changes in the temperature localization phenomenon and topological properties. Thus in our calculations in main text, we assume *P*_1_ = *P*_3_ and define *R* = *P*_2_ / *P*_3_ to carry on the research. Here, to systematically examine the effect of *P*_1_, we perform additional calculations with topological band theory. When *P*_1_ is made larger (say *P_1_* = 2*P_3_* and *P_1_* = 5*P_3_*), steady-state eigenvectors also demonstrate the characteristic of localization to the right (*R*<1) and left (*R*>1). When *R* = 1, unlike uniform distribution when *P*_1_ = *P*_3_, steady-state eigenvectors demonstrate local unevenness in adjacent sites. However, the overall distribution pattern does not show any localization in a particular direction. The larger *P*_1_ is, the more obvious this local unevenness becomes. This local unevenness is also manifested when *R* ≠ 1, e.g. site 12 and 13 when *R* = 0.5 and site 1 and 2 when *R* = 2. Conversely, if *P*_1_ is made smaller (*P_1_* = 0.5*P_3_* and *P_1_* = 0.2*P_3_*), the results are similar. The steady-state eigenvectors also demonstrate the characteristic of localization to the right (*R*<1) and left (*R*>1). And smaller *P*_1_ causes local unevenness in adjacent sites. To sum up, either a larger or a smaller *P*_1_ will not have an impact on the relationship between the localization direction and skewness *R*. The main impact lies in the local unevenness and the temperature deviation between adjacent sites may be strengthened.


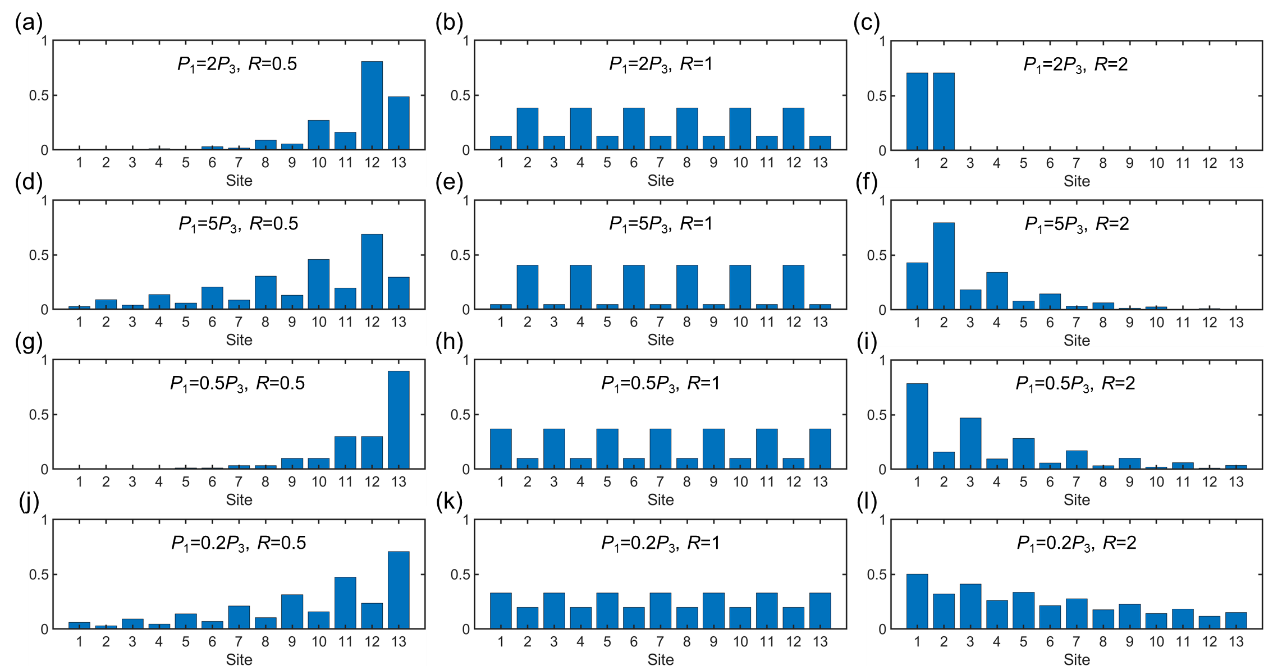


FIG. S5. Steady-state eigenvectors of thermal RPS chain under different values of *P*_1_. (a-f) Steady-state eigenvectors when *P*_1_ is larger (*P_1_* = 2*P_3_* and *P_1_* = 5*P_3_*) (*s* = 13) (g-l) Steady-state eigenvectors when *P*_1_ is smaller (*P_1_* = 0.5*P_3_* and *P_1_* = 0.2*P_3_*) (*s* = 13).

# **Supplemental Note Ⅴ: Band Structure Analysis of Thermal RPS Chain with Diffusion coefficients**

Taking into account the effects of diffusion coefficient *D_j_* and active transport coefficient *P_j_*, the Hamiltonian of thermal RPS chain in *k*-space is

$\text{H}\left(k\right)=\left(-i\right)\left[\begin{matrix} \left(2D_{1}+D_{2}+D_{3}-P_{3}+P_{2}\right)-\left(D_{1}-P_{1}\right)e^{ik}-\left(D_{1}+P_{1}\right)e^{-ik} & -\left(D_{3}-P_{3}\right)-\left(D_{2}+P_{2}\right)e^{ik} \\ -\left(D_{3}+P_{3}\right)-\left(D_{2}-P_{2}\right)e^{-ik} & D_{2}+D_{3}-P_{2}+P_{3} \end{matrix}\right]$(S13)

Then discretize the BZ and calculate the eigenvalues *λ* of the Hamiltonian. Fig. S6 illustrates the band structure of thermal RPS chain under *R* = 1. The value of diffusion coefficients is 0.5, 1 and 2 respectively. When *D_j_* < *P_j_*, the two bands of real parts open while the two bands of imaginary parts close; when *D_j_* > *P_j_*, the two bands of imaginary parts open while the two bands of real parts close; when *D_j_* = *P_j_*, the bands of both real and imaginary parts are exactly closed.


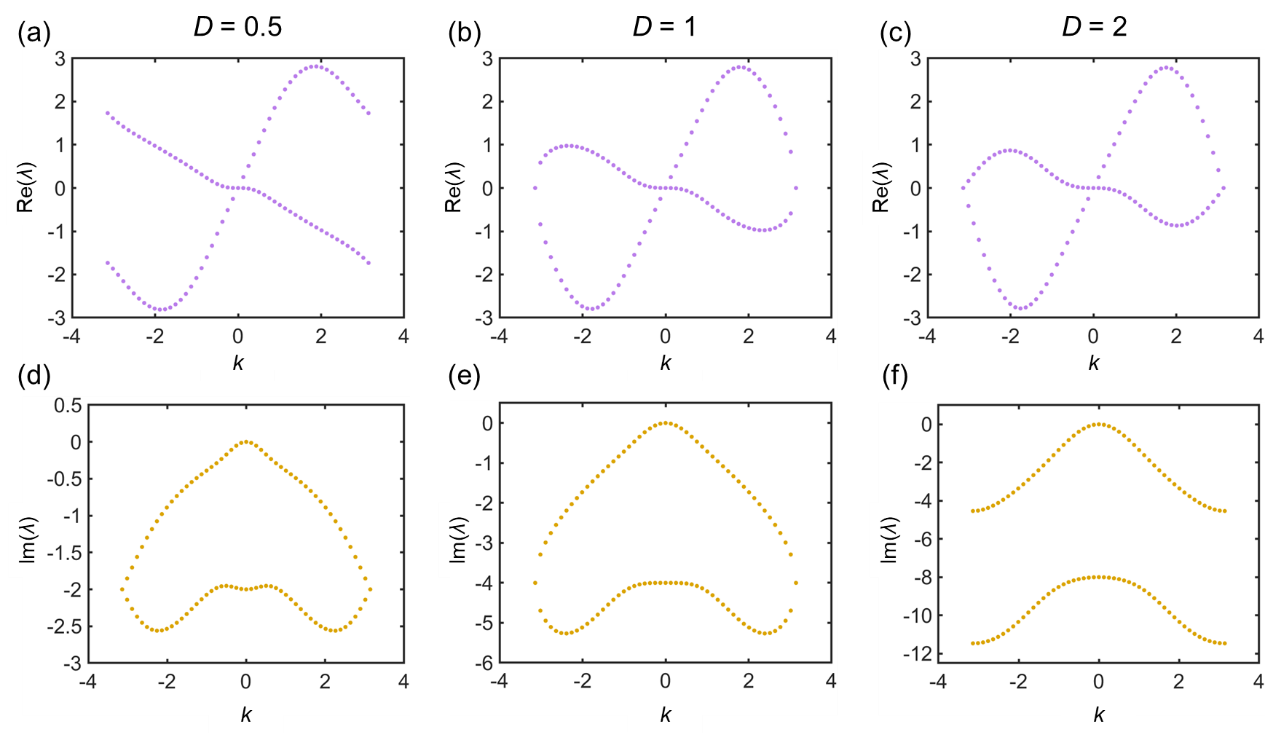


FIG. S6. Band structure of thermal RPS chain in BZ (*R* = 1). (a-c) The real part of eigenvalues of **H** under different diffusion coefficients *D* = 0.5, 1 and 2. (d-f) The imaginary part of eigenvalues of **H** under different diffusion coefficients *D* = 0.5, 1 and 2.


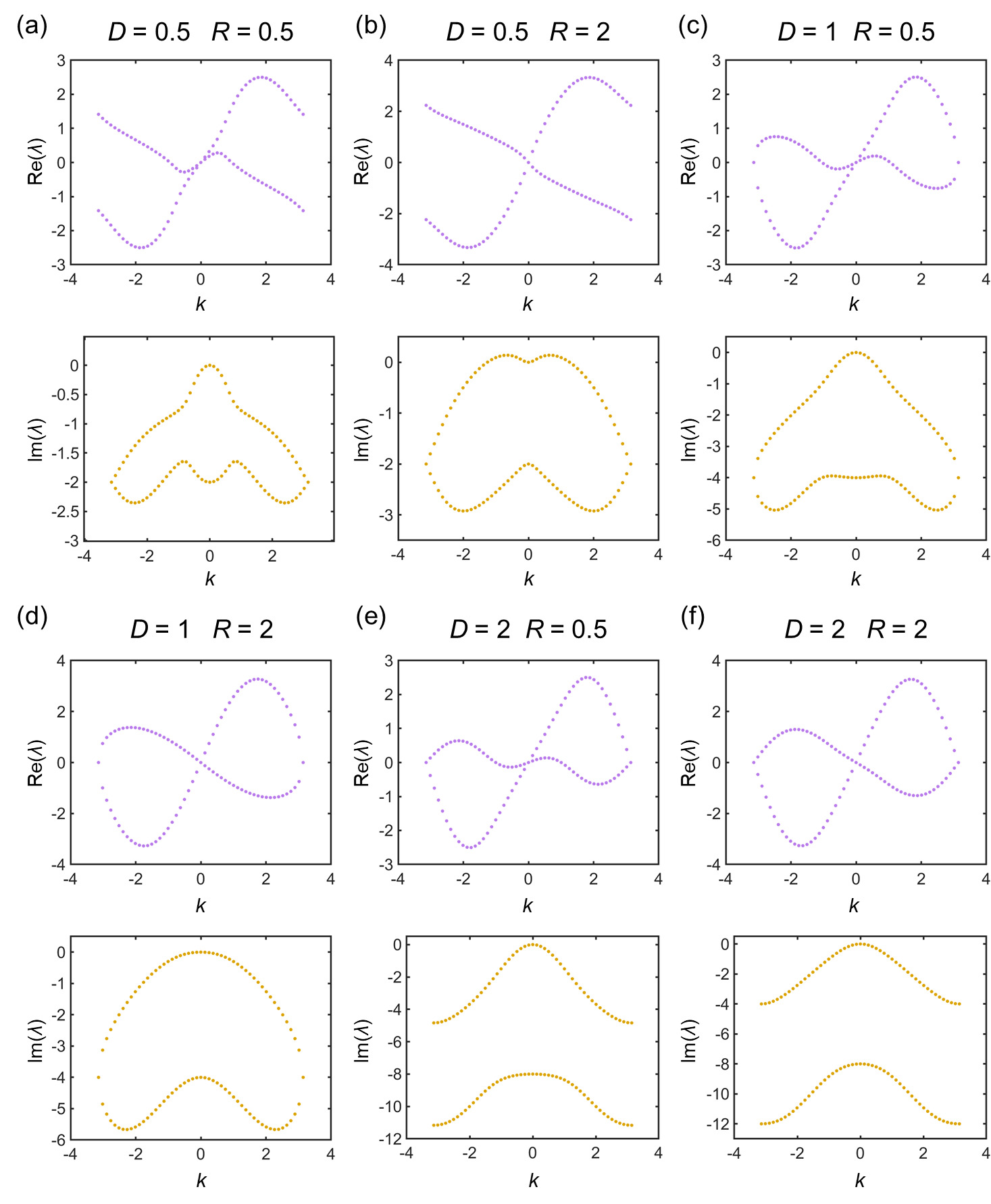


FIG. S7. Band structure of thermal RPS chain in BZ. (a-f) The real part and imaginary part of eigenvalues of **H** under different combinations of parameters. The purple dots represent the real part of the eigenvalue, while the yellow dots represent the imaginary part of the eigenvalue.

Meanwhile, Fig. S7 illustrates the band structures under a more complex *D_j_* - *P_j_* relationship (*R* ≠ 1), and it can be observed that the opening and closing of bands are consistent with the results discussed above. Therefore, the asymmetric relationship in the RPS chain, that is, the values of *R*, does not affect this band structure phenomenon.

Furthermore, we analyze the GBZ curves in the complex plane by incorporating both diffusion coefficient *D_j_* and active transport coefficient *P_j_*. Fig. S8(a) illustrates the GBZ curves under different *D* (*D_1_* = *D_2_* = *D_3_* = *D*) at fixed *R* = 1. All GBZ curves pass through the point (1, 0) in the complex plane. As *D* increases, the GBZ covers a broader region, yet remains entirely confined within the unit circle. Fig. S8(b) illustrates the GBZ curves under different *R* with fixed *D* = 1. Consistent with the simplified case of *D* = 0, when *R* < 1, the corresponding GBZ lies entirely outside the GBZ for *R* = 1, while for *R* > 1, it lies entirely inside the GBZ for *R* = 1.


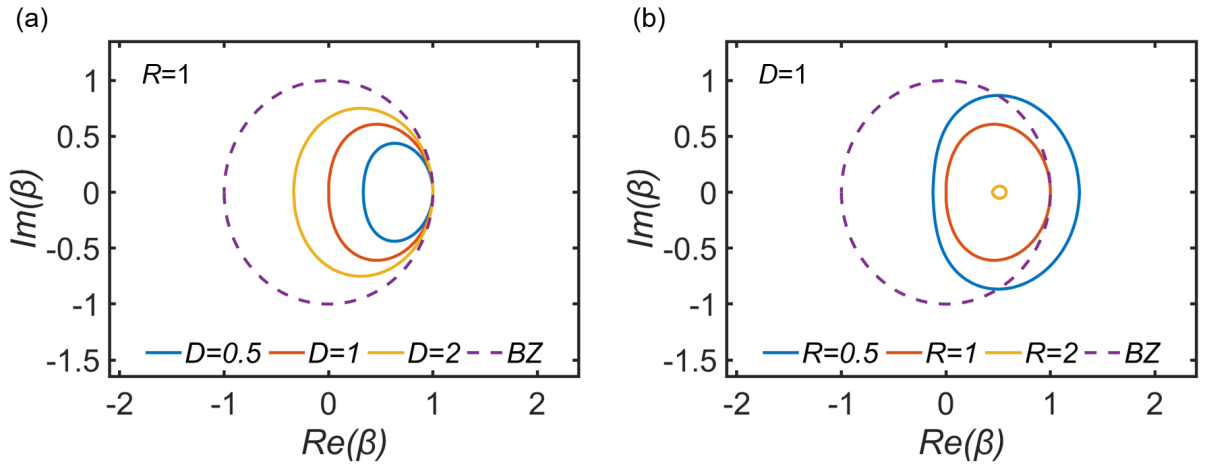


FIG. S8. GBZ of the thermal RPS chain with different combinations of parameters. (a) GBZ curves under *D* = 0.5, 1 and 2 with *R* = 1. (b) GBZ curves under *R* = 0.5, 1 and 2 with *D* = 1. BZ curve is represented by the purple dotted line.


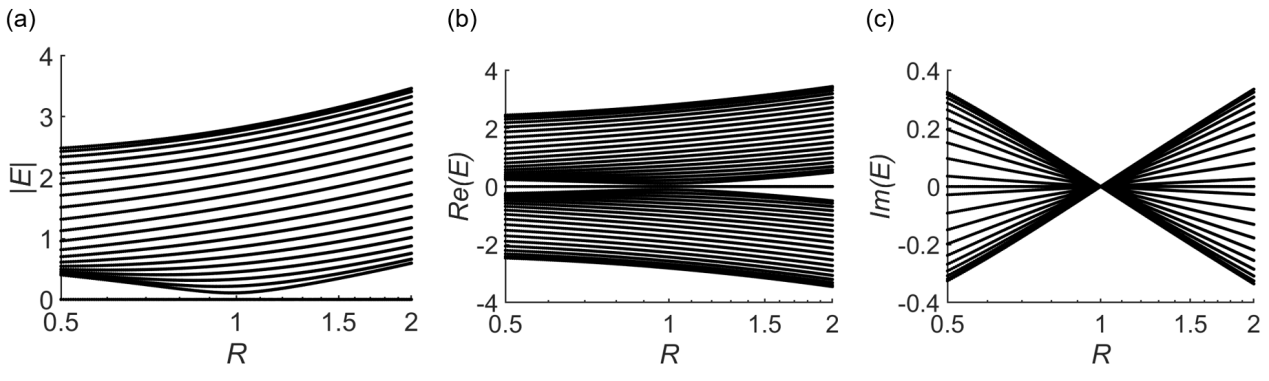


FIG. S9. Numerical spectra of an open thermal RPS chain with *S* = 41. (a) |E| as functions of *R*. (b), (c) The real and imaginary part of *E*.
